# Down-regulated miR-22 as predictive biomarkers for prognosis of epithelial ovarian cancer

**DOI:** 10.1186/s13000-014-0178-8

**Published:** 2014-09-26

**Authors:** Wei-na Wan, Yu-qin Zhang, Xue-mei Wang, Yan-jun Liu, Yi-xia Zhang, Yan-hong Que, Wen-jing Zhao, Ping Li

**Affiliations:** Department of Ultrasound, First Affiliated Hospital of China Medical University, No. 155 Nanjing North Street, Shenyang, Liaoning 110001 China

**Keywords:** miR-22, Biomarkers, Prognosis, Epithelial ovarian cancer

## Abstract

**Background:**

Recent studies have demonstrated that microRNA-22 (miR-22) was deregulated in many types of cancers and was involved in various cellular processes related to carcinogenesis. However, the clinical significance and prognostic value of miR-22 in epithelial ovarian cancer (EOC) haven’t been investigated.

**Methods:**

109 pairs of fresh EOC tissue and matched adjacent normal tissue specimens were collected between May 2007 and March 2013. Real-time quantitative RT-PCR assay was performed to evaluate the expression levels of miR-22. The chi-square test was used to assess miR-22 expression with respect to clinicopathological parameters. The survival curves of the patients were determined using the Kaplan-Meier method and Cox regression, and the log-rank test was used for statistical evaluations.

**Results:**

miR-22 expression in EOC tissues was significantly lower than that in matched normal adjacent tissues (mean ± SD: 1.944 ± 1.026 vs. 4.981 ± 1.507, *P* < 0.0001). Low miR-22 expression level was correlated with FIGO stage (*P* = 0.006), tumor grade (*P* = 0.03), and lymph node metastases (*P* = 0.01). Kaplan-Meier analysis with the log-rank test indicated that low miR-22 expression had a significant impact on overall survival (44.4% vs. 64.5%; *P* = 0.005) and progression-free survival (23.5% vs. 52.6%; *P* = 0.004).

**Conclusions:**

Our data demonstrated that the expression of miR-22 was downregulated in EOC, and associated with overall survival as well as progression-free survival, suggesting that miR-22 could serve as an efficient prognostic factor for EOC patients.

**Virtual slides:**

The virtual slide(s) for this article can be found here: http://www.diagnosticpathology.diagnomx.eu/vs/13000_2014_178

## Background

Ovarian cancer, particularly epithelial ovarian cancer (EOC), which accounts for 90% of all ovarian cancers, continues to be the leading cause of death among gynaecological malignancies [1]. Furthermore, the majority of cases are diagnosed with ovarian cancer at later stages [2]. Stage at diagnosis, maximum residual disease following cytoreductive surgery, and performance status are the three major prognostic factors for ovarian cancer. Using a multimodality approach to treatment, including aggressive cytoreductive surgery and combination chemotherapy, five-year survival rates are as follows: Stage I (93%), Stage II (70%), Stage III (37%), and Stage IV (25%) [3]. Therefore, the identification of novel diagnostic and prognostic biomarkers for treatment response is eagerly desired.

MicroRNAs (miRNAs) are small non–protein-coding RNAs that regulate gene expression post-transcriptionally by interacting with partially complementary target sites in mRNAs, either inducing their degradation or impairing their translation. miRNAs are implicated in several diseases and cellular functions, including apoptosis, differentiation, as well as proliferation. Aberrant miRNA expression levels are associated with tumorigenesis, progression, and metastases, acting as oncogenes and/or tumor suppressors [4-7]. Recently, microRNA-22 (miR-22) has been shown to be deregulated in some types of cancers, such as overexpression in prostate cancer and downregulation in breast cancer, cholangiocarcinoma, hepatocellular carcinoma (HCC), gastric cancer, lung cancer, colorectal cancer (CRC), and multiple myeloma [6,8-14]. Therefore, we hypothesized that miR-22 might play a role in EOC. Hence, in the present study, we focus on the expression and clinical significance of miR-22 in EOC.

## Methods

### Patients and tissue samples

This study was approved by the Research Ethics Committee of the First Affiliated Hospital of China Medical University. Written informed consent was obtained from all patients. All specimens were handled and made anonymous according to the ethical and legal standards. 109 pairs of fresh EOC tissue and matched adjacent normal tissue specimens were collected from patients who underwent surgery between May 2007 and March 2013 in the First Affiliated Hospital of China Medical University. The fresh tissue specimens were collected and immediately placed in liquid nitrogen and then stored at-80°C until the isolation of RNA. Clinico-pathological data including age, pathological stage, histology, lymph node metastases and tumor grade were collected. Patient characteristics are shown in Table 1. None of the patients recruited in this study had undergone preoperative chemotherapy or radiotherapy. The duration of follow-up was calculated from the date of surgery to death or last follow-up, and patients were excluded if they had incomplete medical records or inadequate follow-up. All patients had a follow up > 1 year. Disease progression was defined by either CA125 ≥ 2 × nadir value on two occasions, documentation of increase or new lesions on CT-scan or death [15]. Patient’s conditions were staged according to the criteria of the International Federation of Gynecology and Obstetrics (FIGO).

**Table 1:** **Correlations of miR-22 expression with the clinicopathological features of EOC**

**Variables**	**n**	**miR-22 expression level**		**P value**
		**Low**	**High**	
Age, y				
<55	46	16	30	0.29
≥55	63	39	24	
FIGO stage				
I–II	61	17	44	0.006
III–IV	48	38	10	
Histology				
Serous	71	34	37	0.65
Nonserous	38	21	17	
Residual tumor size (cm)				
<1.0	79	37	42	0.09
≥1.0	30	18	12	
Grade				
Well	31	5	26	0.03
Moderate	43	23	20	
Poor	35	27	8	
Lymph node metastases				
Negative	81	34	47	0.01
Positive	28	21	7	
Serum CA125				
<319	56	24	32	0.12
≥319	53	31	22	

### RNA extraction and quantitative RT-PCR

Total RNA was isolated from frozen specimen by homogenizing tissue in Trizol reagent (Invitrogen, Carlsbad, CA, USA), according to the manufacturer’s instructions. The purity and concentration of RNA were determined using NanoDrop 1000 spectrophotometer (Thermo Scientific, Wilmington, DE, USA). The differentially expressed amount of the miR-22 was validated in triplicate by quantitative reverse-transcription polymerase chain reaction (qRT-PCR). Briefly, 2 ug of RNA was added to RT reaction, and then, the cDNA served as the template for amplification of PCR with sequence-specific primers (Sangon Biotech, Shanghai, China) using SYBR PrimeScript miRNA RT-PCR kit (Takara Biotechnology Co. Ltd, Dalian, China) on the 7500 Real-Time PCR systems (Applied Biosystems, Carlsbad, CA, USA). The PCR cycling profile was denatured at 95°C for 30 s, followed by 40 cycles of annealing at 95°C for 5 s, and extension at 60°C for 34 s. Small nucleolar RNA U6 was used as an internal standard for normalization. The cycle threshold (C_T_) value was calculated. The 2^-ΔCT^ (ΔC_T_ = C_TmiR22_-C_TU6 RNA_) method was used to quantify relative amount of miR-22.

Real-time PCR primers: miR-22: F: 5′-ACACTCCAGCTGGGTTCGACGGTCAACTTC-3′.

R: 5′- CTCAACTGGTGTCGTGGAGTCGGCAATTCAGTTGAGACAGTTCT-3′; U6: F: 5′-GCGCGTCGTGAAGCGTTC-3′; R: 5′-GTGCAGGGTCCGAGGT-3′.

### Statistical analysis

Statistical analysis was conducted using the SPSS 18.0 for Windows (SPSS Inc., Chicago, IL, USA). The chi-square test was used to assess miR-22 expression with respect to clinicopathological parameters. The survival curves of the patients were determined using the Kaplan-Meier method and Cox regression, and the log-rank test was used for statistical evaluations. Univariate Cox regression was performed on each clinical covariate to examine its influence on patient survival. Final multivariate models were based on step-wise addition. A Wald statistic of *P* < 0.05 was used as the criterion for inclusion in final multivariate models. Data were expressed as the mean and standard deviation and analyzed using one-way analysis of variance. *P* < 0.05 was considered to indicate a significant difference.

## Results

### miR-22 is downregulated in EOC

**Figure 1:**
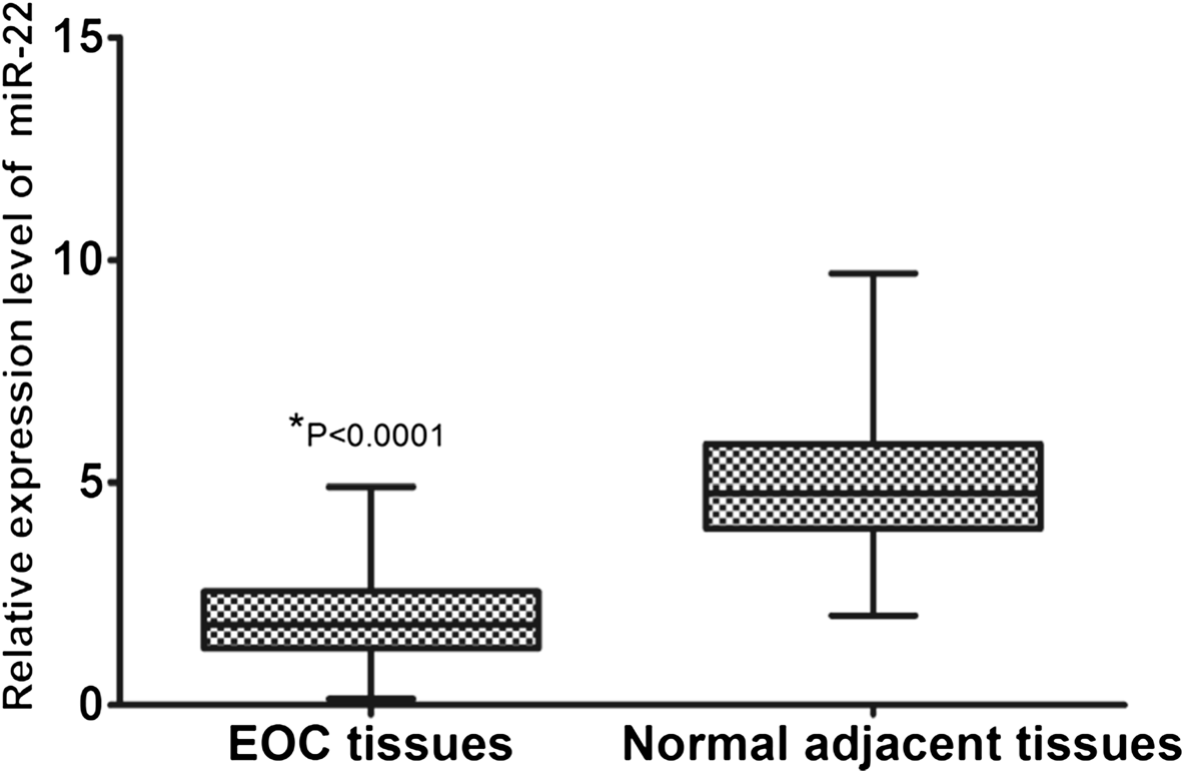
**miR-22 expression in 109 pairs of EOC tissue and matched adjacent normal tissue specimens by qRT-PCR.** All data were normalized to U6B.

qRT-PCR was performed to detect the differential expression of miR-22 in 109 pairs of EOC tissues and matched normal adjacent tissues. As a result, miR-22 expression in EOC tissues was significantly lower than that in matched normal adjacent tissues (mean ± SD: 1.944 ± 1.026 vs. 4.981 ± 1.507, *P* < 0.0001, shown in Figure 1).

### Reduced expression of microRNA-22 is associated with advanced clinicopathologic characteristics of patients with EOC

The 109 patients with EOC were classified into two groups according to the median expression level of miR-22. Of the 109 patients with EOC, 55 were placed in the low miR-22 expression group and 54 were placed in the high miR-22 expression group. The associations between clinicopathologic features and miR-22 expression were summarized in Table 1. Low miR-22 expression level was correlated with FIGO stage (*P* = 0.006), tumor grade (*P* = 0.03), and lymph node metastases (*P* = 0.01). However, low miR-22 expression was not associated with other clinicopathological factors of EOC patients, including age, histology, residual tumor size, as well as serum CA125 (all *P* > 0.05, shown in Table 1).

### Association between miR-22 expression and survival in EOC patients

**Figure 2:**
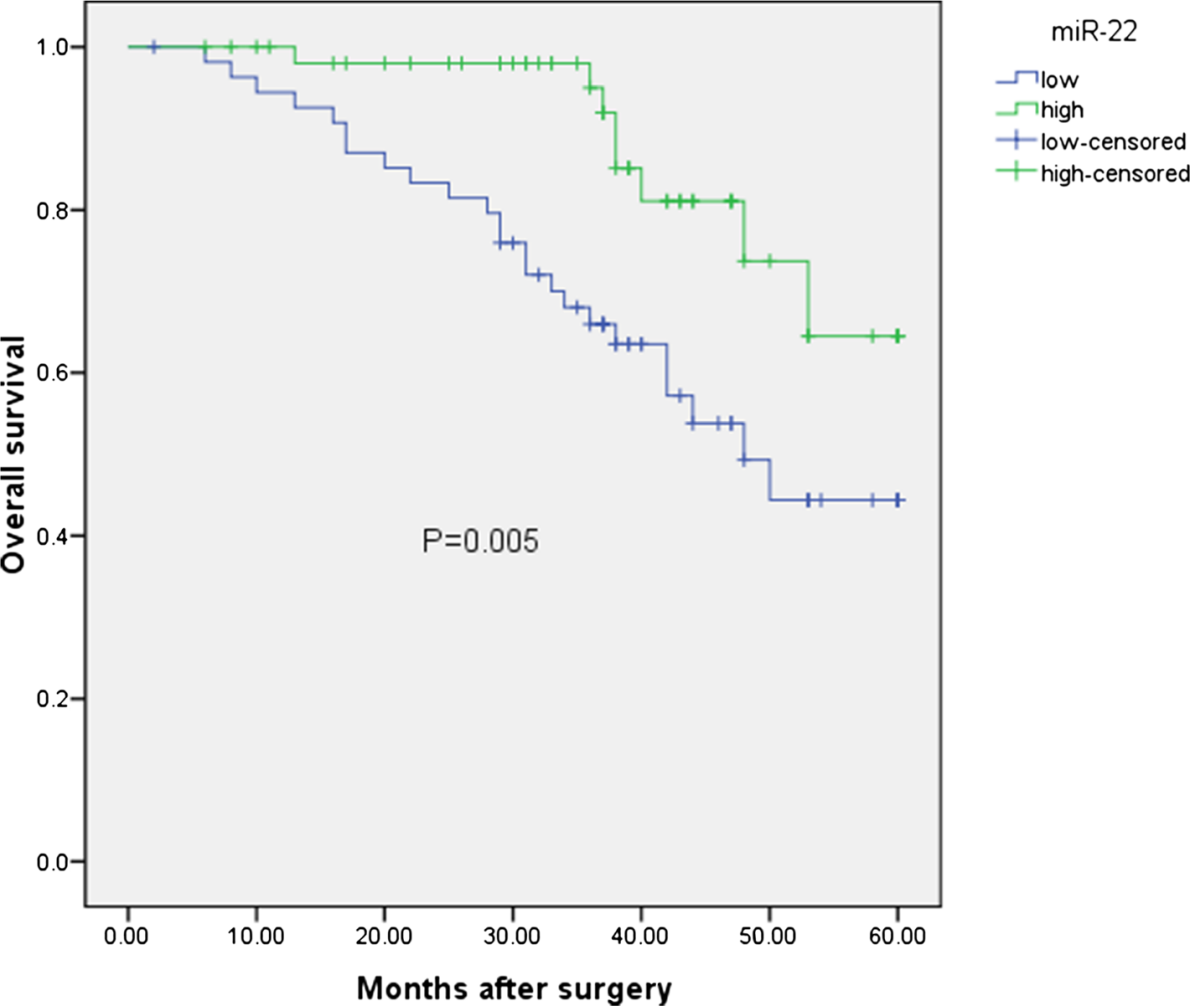
**The expression of miR-22 in relation to overall survival in the patients with EOC.**

**Figure 3:**
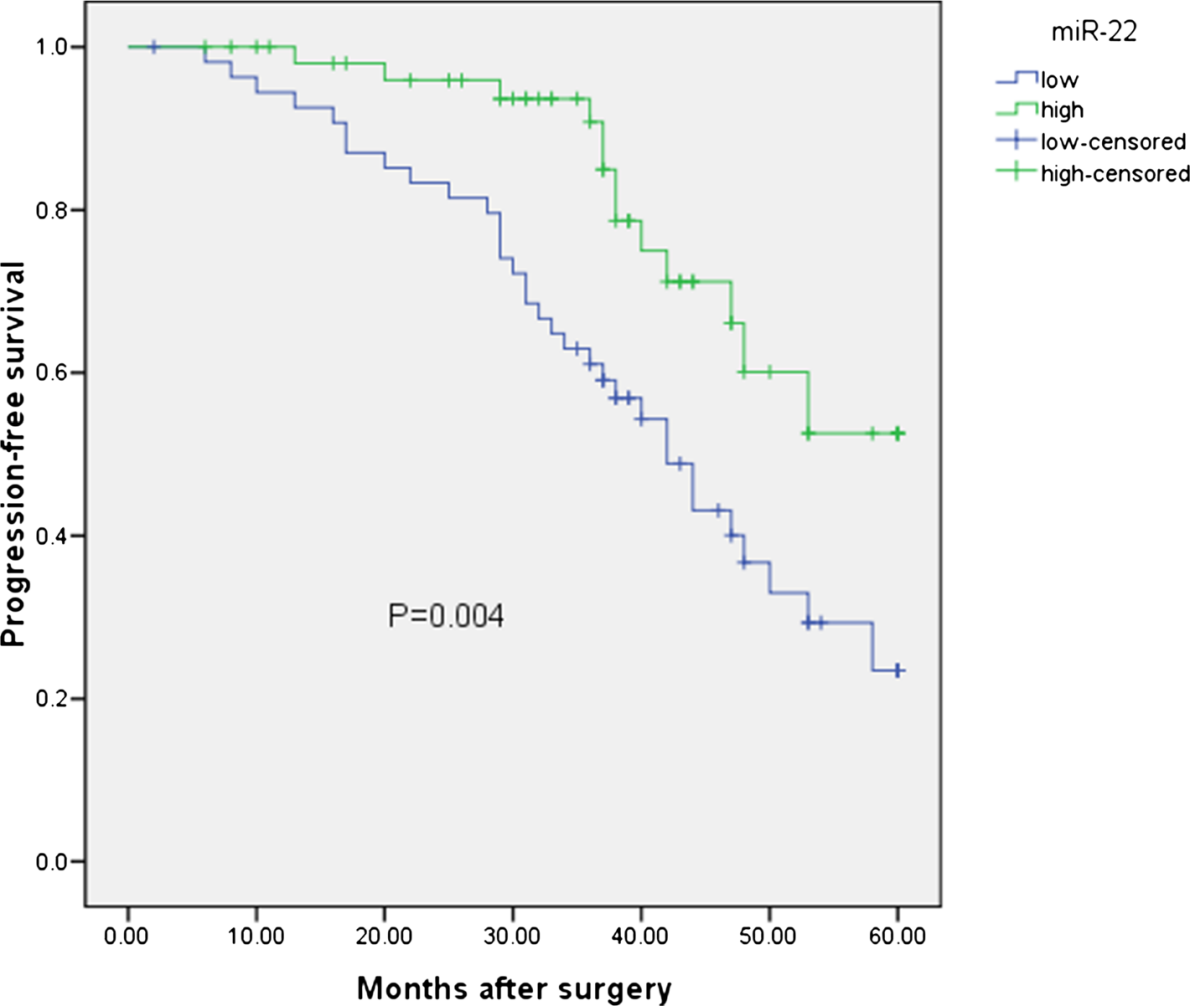
**The expression of miR-22 in relation to progression-free survival in the patients with EOC.**

Kaplan-Meier analysis with the log-rank test indicated that low miR-22 expression had a significant impact on overall survival (44.4% vs. 64.5%; *P* = 0.005; shown in Figure 2) and progression-free survival (23.5% vs. 52.6%; *P* = 0.004; shown in Figure 3). Univariate and multivariate analyses were utilized to evaluate whether the miR-22 expression level and various clinicopathological features were independent prognostic parameters of EOC patient outcomes. Multivariate analysis revealed that miR-22 expression level was an independent prognostic factor for overall survival (HR = 2.552, 95% CI: 1.961-9.763; *P* = 0.007), as well as progression-free survival (HR = 2.341, 95% CI: 2.021-11.672; *P* = 0.005) of EOC patients (shown in Table 2).

**Table 2:** **Multivariate cox proportional hazard model analysis of overall survival and progression-free survival in 109 patients with ovarian cancer**

	**Overall survival**			**Progression-free survival**		
	**Hazard ratio**	**95% Confidence interval**	**P value**	**Hazard ratio**	**95% Confidence interval**	**P value**
Age	0.942	0.628-2.093	0.272	0.872	0.389-1.845	0.356
FIGO stage	3.893	1.267-9.273	0.031	4.342	1.925-10.287	0.009
Histology	1.272	0.287-3.532	0.783	1.372	0.228-2.091	0.691
Residual tumor size	2.382	0.871-6.281	0.086	2.192	0.754-3.002	0.072
Grade	3.091	2.004-8.921	0.021	3.971	2.716-9.021	0.012
Lymph node metastases	1.872	1.022-9.655	0.031	3.911	2.241-12.891	0.009
Serum CA125	3.297	0.652-7.271	0.051	2.467	0.811-6.188	0.068
MiR-22 expression level	2.552	1.961-9.763	0.007	2.341	2.021-11.672	0.005

## Discussion

Many studies have intensely focused on the function of altered miRNA expression in human cancer in recent years [16]. Some miRNAs in cancer cells could play a role as oncogenes to inhibit the expression of tumor suppressors [17]. Moreover, the physiological and pathological roles of miRNAs have also been demonstrated in most tumor types and miRNAs may play an important role in the diagnosis, prognosis, and treatment of cancer [7,18]. Therefore, the correlations between miRNAs and cancers have become a focus of cancer studies. Previously, researchers have found that disregulation of several miRNAs are associated with the prognosis in patients with cancer, suggesting that miRNA expression level detection might become a potential biomarker of prognosis in cancer [19-23].

miR-22 is located at a fragile cancer-relevant genomic region in chromosome 17 (17p13.3), and is mapped to an exon of the C17orf91 gene [24]. This miRNA plays unique roles in specific cell types. For example, it regulates PPARalpha and BMP7 signaling pathways in human chondrocytes [25], and the differentiation of a monocyte cell line [26]. Recent studies have demonstrated that miR-22 is deregulated in many types of cancers and is involved in various cellular processes related to carcinogenesis, including cell growth, apoptosis, motility, and cell cycle. Zhang et al. indicated that miR-22 was downregulated in HCC and had considerable potential in identification of the prognosis [10]; Xiong et al. found that miR-22 was also downregulated in breast cancer, and it suppressed breast cancer development through directly targeting oestrogen receptor α (ERα) and downstream signaling [11]; Wang et al. offered the convincing evidence that the reduced expression of miR-22 was significantly associated with malignant development of gastric cancer and may be a novel prognostic marker [8]; Yamakuchi et al. found that miR-22 expression in human colon cancer was lower than in normal colon tissues, and it might have an anti-angiogenic effect in this cancer [27]; Ling et al. observed the downregulation of miR-22 in lung cancer tissues and lung cancer cell lines, and also suggested that miR-22 might exhibit excellent anti-lung cancer activity in vitro and in vivo [28]. However, miR-22 expression was suggested to be upregulated in prostate cancer, and its upregulation potentiated phosphatidylinositol 3-kinase-Akt pathway activation [29]. These controversial results of miR-22 in cancer development may reflect the diverse roles of miR-22 in different types of cancers.

Previously, Li et al. found that there was a negative correlation between miR-22 expression and the metastatic potential in ovarian cancer cells. Furthermore, both gain-of-function and loss-of-function studies displayed an inhibitory effect of miR-22 on cell migration and invasion in vitro without significantly affecting cell viability and apoptosis. Subsequent bioinformatics analysis revealed that miR-22 might regulate multiple pro-metastatic genes, which could provide an explanation to the inhibitory effects of miR-22 on cell migration and invasion. Taken together, their findings suggested that miR-22 might be involved in inhibiting ovarian cancer metastasis [30]. However, the clinical significance and prognostic value of miR-22 in EOC haven’t been investigated. Hence, in the present study, we focused on the expression and clinical significance of miR-22 in EOC. We found that miR-22 expression in EOC tissues was significantly lower than that in matched normal adjacent tissues. Then the relationships of the miR-22 with various clinical features of EOC were analyzed. We found that low miR-22 expression level was correlated with FIGO stage, tumor grade, and lymph node metastases, suggesting that miR-22 might be involved in the carcinogenesis and metastasis of EOC. Furthermore, Kaplan-Meier analysis with the log-rank test indicated that low miR-22 expression had a significant impact on overall survival and progression-free survival. Univariate and multivariate analyses were utilized to evaluate whether the miR-22 expression level and various clinicopathological features were independent prognostic parameters of EOC patient outcomes. Multivariate analysis revealed that miR-22 expression level was independent prognostic factors for overall survival, as well as progression-free survival of EOC patients, indicating that low miR-22 level was a promising non-invasive biomarker for prognosis of patients with EOC.

## Conclusion

In conclusion, our data demonstrated that the expression of miR-22 was downregulated in EOC, and associated with overall survival as well as progression-free survival, suggesting that miR-22 could serve as an efficient prognostic factor for EOC patients.
